# Feasibility, Safety, and Patient Satisfaction of Transurethral Bladder Tumor Resection in an Outpatient Setting

**DOI:** 10.1002/cnr2.70120

**Published:** 2025-03-12

**Authors:** Lucas Strahl, Hendrik Borgmann, Julian Peter Struck, Johannes Salem, Timur H. Kuru

**Affiliations:** ^1^ CUROS Urologisches Zentrum Cologne Germany; ^2^ Klinik für Urologie und Kinderurologie, Universitätsklinikum Brandenburg an der Havel Brandenburg an der Havel Germany

**Keywords:** bladder cancer, healthcare efficiency, outpatient TURB, patient satisfaction, surgical outcomes

## Abstract

**Introduction:**

Transurethral resection of the bladder (TURB) is a common urological procedure, typically performed in an inpatient setting. This study aims to investigate safety, quality, and patient satisfaction aspects of TURB in an outpatient setting, reflecting the emerging strategy of outpatientization of surgical procedures in the German healthcare system.

**Methods:**

We retrospectively analyzed a cohort of 100 patients who underwent outpatient TURB. The standard procedure was day surgery under general anesthesia and dismissal with or without a urinary catheter. The analysis focused on postoperative complications, resection quality, recurrence within 6 months, and patient satisfaction. Data was collected from electronic medical records and patient interviews and analyzed using descriptive and multivariate statistics.

**Results:**

The cohort consisted primarily of male patients (79%) with a median age of 70 years. The average surgery time was 11.3 min with a mean of 1.6 tumors resected. Histopathological findings leaned toward superficial bladder tumors with a mean recurrence rate of 11.6%. Postoperative complications were mostly mild, with only 5% of patients suffering from complications rated as Clavien–Dindo ≥ 2 and no complications of grade ≥ 4. High levels of patient satisfaction were reported, with 83% preferring outpatient TURB to inpatient treatment for future surgery.

**Conclusion:**

Outpatient TURB appears to be a safe and effective alternative to inpatient surgery for selected patients with bladder tumors ≤ 4 cm, offering comparable surgical and oncological outcomes while enhancing patient satisfaction and reducing healthcare system burden.

## 1 Introduction

Bladder cancer (BC) is the fifth most diagnosed cancer in Germany [1]. When bladder cancer is suspected, transurethral resection of the bladder (TURB) is indicated for both, diagnosis and treatment [2]. Consequently, TURB is the most frequently performed urological intervention in an inpatient setting in German hospitals, it was performed over 120.000 times in 2021 [3] and the expenses for the treatment of bladder cancer in German hospitals exceeded 630 million Euros in 2020 [4].

The concept of “outpatientization” describes the transfer of certain inpatient surgical procedures into an outpatient setting. It has emerged as a strategy to address the future challenges of the German Health Care System, according to the German Federal Ministry of Health [5].

This study aimed to evaluate safety aspects, quality aspects, and patient satisfaction of outpatient TURB. We analyzed a consecutive cohort of patients presenting for TURB in an office‐based urology clinic. Infectious and non‐infectious complications, resection quality, as well as recurrence rate within 6 months were analyzed. Furthermore, we conducted a patient survey to investigate patient satisfaction aspects.

While outpatient TURB offers potential benefits such as lower healthcare costs and reduced hospital‐acquired infection risks, it also necessitates precise patient selection criteria and robust follow‐up protocols to manage any complications effectively. Our study aims to contribute to this topic by examining outcomes and patient‐reported experiences in a real‐world outpatient setting.

## 2 Methods

We analyzed a cohort of 100 patients retrospectively, having undergone TURB in an outpatient setting from 2020 to 2023 at Klinik LINKS VOM RHEIN, an office‐based private hospital located in Cologne, Germany.

Inclusion criteria were patients with bladder tumors measuring < 4 cm identified during cystoscopic examination and patients indicated for repeat TURB (Re‐TURB) due to clinical factors such as high‐grade histology, absence of detrusor muscle in the initial specimen, or incomplete initial resection. Exclusion criteria were tumors ≥ 4 cm in size, an ASA score ≥ 4, or an insufficient social support network, which made transport to and from the private clinic unmanageable. Tumors larger than 4 cm were excluded due to the increased complexity of resection and higher risk of perioperative complications.

The intervention was carried out by two board‐certified senior urologists using standardized surgical techniques and postoperative care protocols, ensuring consistency across patient treatments. The patients were admitted to the private hospital by the specialist physicians of the practice network.

The standard procedure was day surgery under general anesthesia and perioperative antibiotic treatment. The surgical strategy of TURB was guideline‐based: bladder tumors were resected en‐bloc or in fractions using bipolar current. Narrow‐band imaging (NBI) was used to enhance tumor visualization, improving contrast between normal urothelium and neoplastic tissue, potentially contributing to higher resection quality and reduced recurrence rates. Specimens from different resection fractions (e.g., tumor margin, tumor base) were submitted individually.

After surgery, patients underwent circulatory monitoring and bladder irrigation. Given an uneventful surgery, circulation parameters within range, and absence of clinically significant macrohematuria, patients were discharged around 2 h after surgery.

The decision for discharge with or without a urinary catheter was dependent on resection depth and the level of macrohematuria. For patients eligible for single instillation (SI) according to EAU guidelines (preferably low‐grade pTa tumor and/or primary tumor), the patient was informed for the SI to be administered in the responsible doctor's practice within 24 h after surgery.

Regarding postoperative follow‐up, all patients were provided with an information sheet outlining typical harmless complications associated with TURB, such as bleeding, dysuria, and burning, which are classified as Clavien–Dindo grade ≤ 2. Additionally, lifestyle recommendations were included to support recovery, including increased water intake, needs‐based pain management with over‐the‐counter analgesics like ibuprofen, and guidelines for physical rest. The information sheet also contained emergency phone numbers for reaching the doctor's office directly or contacting the surgeon after hours. Each patient was scheduled for a follow‐up appointment between 5 and 10 days after surgery at their respective doctor's office. During these appointments, histology results were reviewed, and patients had the opportunity to discuss their recovery and any potential issues.

Data was extracted by reviewing patients' electronic medical records, which included practice notes, medical history, the documentation of pre‐ and postoperative examinations like cystoscopy reports, surgery reports, and pathology reports. Furthermore, we conducted structured phone interviews using a standardized questionnaire with 60 of 100 patients, the remaining 40 patients could not be contacted or did not wish to get interviewed. This approach ensured that data collection was uniform across all participants, minimizing variability in responses and enhancing the reliability of patient‐reported outcomes (see Appendix A for the questionnaire). The median time between surgeries to phone interview was 22 months, ranging from 3 to 39 months.

Statistical analysis was performed using IBM SPSS Statistics V19. The study population was described by standard descriptive statistical measures. For correlation statistics, we performed multivariate analysis, partly with subgroup analysis, for certain target variables like postoperative complications, which were categorized according to Clavien–Dindo classification [6]. Follow‐up period for complications was 90 days. *p* values < 0,05 were defined as statistically significant.

## 3 Results

From January 2021 until January 2023, 100 patients (79 male, 21 female) underwent outpatient TURB. The median age at surgery was 70 years, ranging from 35 to 94 years. The median age of patients diagnosed with bladder cancer was 72 years, ranging from 52 to 94 years. ECOG distribution was as follows: ECOG 0: 21%, ECOG 1: 28%, ECOG 2: 35%, ECOG 3: 15%, and ECOG 4: 1%.

The mean surgery duration was 11.3 min, with a mean of 1.6 tumors resected, ranging from 1 to 6 tumors. The mean tumor size was 10.7 mm, ranging from 3 to 35 mm, with 61% of the tumors being located at the side walls of the bladder.

Exactly 30% of patients were dismissed with a urinary catheter, which was removed after a median of 36 h. Consequently, 70% of patients were dismissed without a urinary catheter.

The histopathological results showed no malignancy in 55% of patients, 45% of patients were diagnosed with bladder cancer (pTa low grade: 55.6%, pTa high grade 26.7%, pT1: 4.4%, pT2: 8.9%, CIS: 4.4%).

**FIGURE 1:**
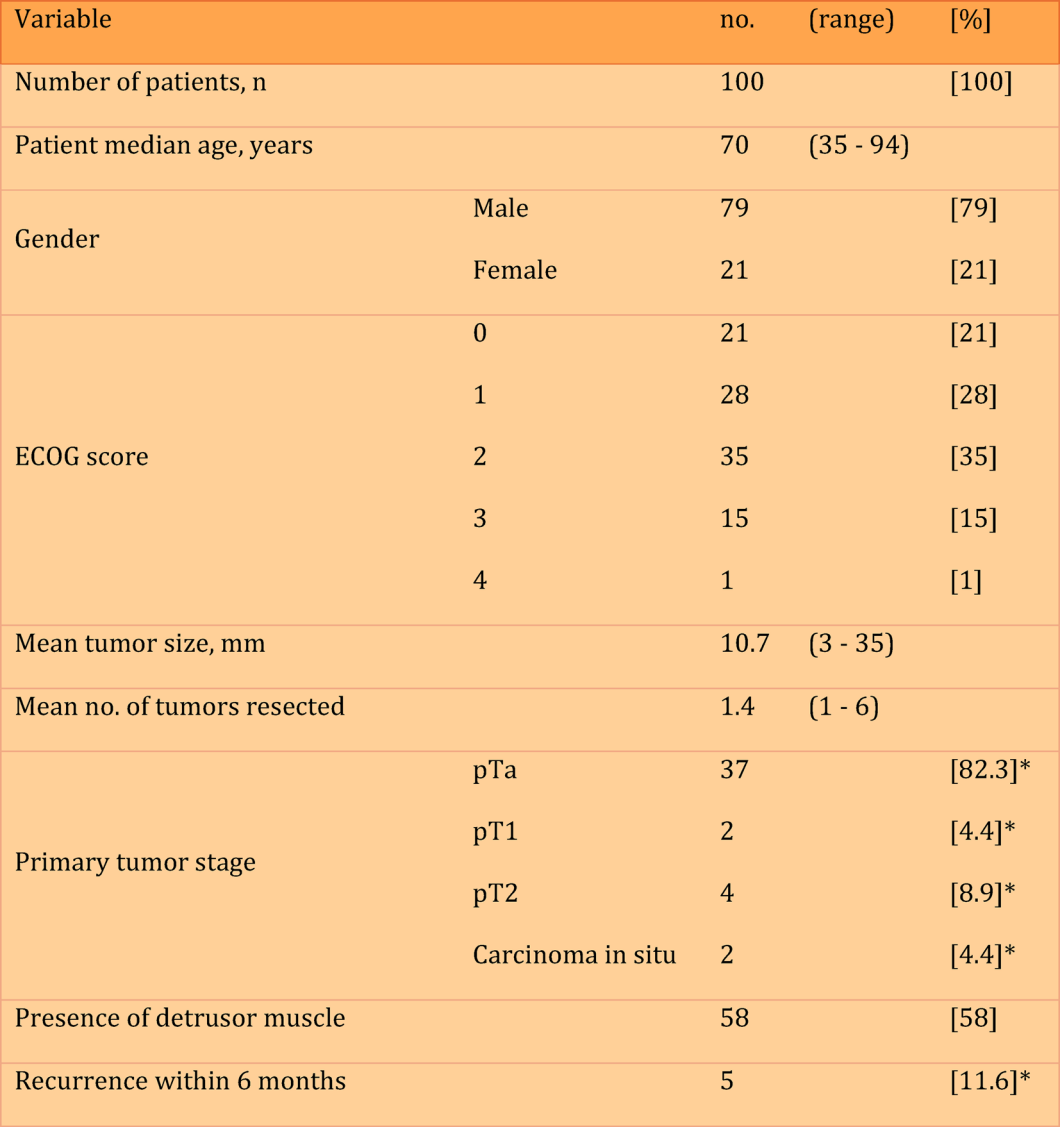
Patient demographics and tumor characteristics. * respective to patients with bladder cancer.

Concerning resection quality, we achieved the presence of detrusor muscle (DM) in the specimen in 58% of patients. Subgroup analysis of patients with tumor classification other than pTa G1 low grade, where EAU guidelines strongly recommend the presence of detrusor muscle to minimize the risk of residual disease and tumor under‐staging, showed a presence of detrusor muscle in 66.7%. All patients necessitating second TURB (according to EAU guidelines) were admitted respectively. Figure 1 provides an overview of the demographic distribution and tumor characteristics of the patient cohort.

**FIGURE 2:**
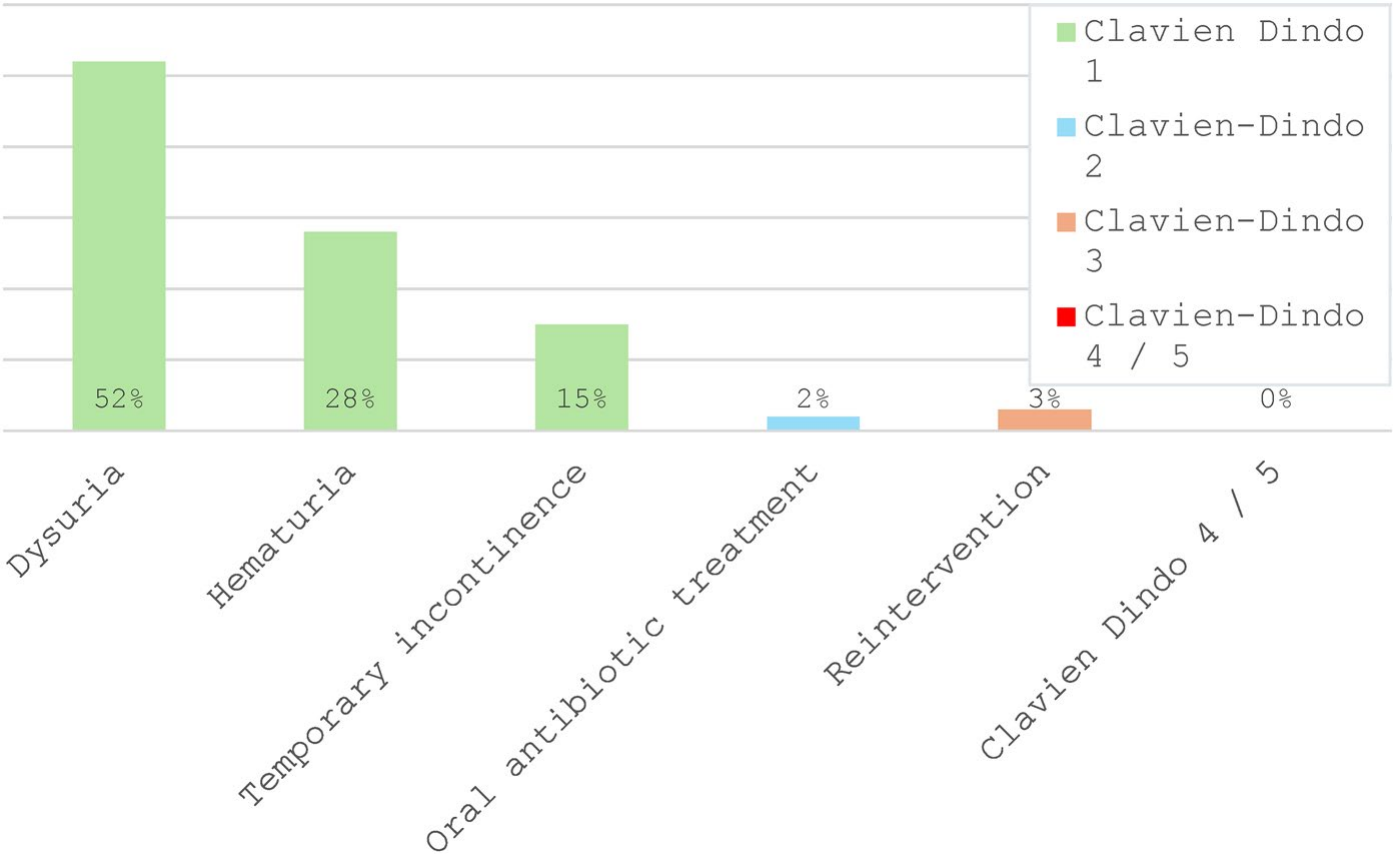
Postoperative complications within 90 days.

Regarding postoperative complications categorized as Clavien Dindo 1, 32 patients (53%) of those interviewed reported mild dysuria, 17 patients (28%) reported mild hematuria, and 9 patients (15%) had temporary urinary incontinence. One patient (1%) required a visit to an external urological emergency department within 24 h after surgery due to acute complications (bladder tamponade with unilateral hydronephrosis). Overall, 5% of patients had complications categorized as Clavien–Dindo ≥ 2: postoperative oral antibiotic therapy was necessary for 2 patients because of urinary tract infection. Surgical reintervention was indicated for 3 patients (one patient with bladder tamponade with unilateral hydronephrosis, two patients with prolonged hematuria). There was no case of blood transfusion or complications Clavien Dindo ≥ 4 necessitating ICU treatment or resulting in death (shown in Figure 2).

Comparing patients dismissed with or without urinary catheter there were more severe complications in the catheter group reflected in a higher Clavien Dindo score (0.53 vs. 0.17, *p* = 0.045).

In multivariate regression analysis, tumor size (Pearson's correlation coefficient: 0.471, *p* = 0.006) and surgery duration (Pearson's correlation coefficient: 0.304, *p* = 0.006) showed a moderate positive correlation with higher complication scores. This suggests that larger tumor sizes and longer surgeries are significantly associated with increased postoperative complications.

Five patients suffered from early recurrence within 6 months (11.6%) (patient 1 and patient 2: pTa G1 low grade > pTa G1 low grade, patient 3: pTa G1 low grade > pTa G3 high grade + CIS; patient 4: pTa G2 high grade > pTa G2 high grade, patient 5: pT1 G3 high grade > pT2a G3 high grade). The mean time to recurrence was 119 days.

Patient satisfaction was very high, with an overall average score of 1.53 on a scale of 1 (very good) to 6 (insufficient). The evaluation assessed three key aspects: the patient consent talk, the surgery itself, and the aftercare. Specifically, patients discharged with a urinary catheter managed well or very well with it. Only 10.7% of patients expressed any fear of postoperative complications (low: 1.8%, moderate: 3.6%, strong: 5.4%), while 89.3% had no such fears. Nearly all patients (91.1%) did not experience feelings of isolation after surgery. Ultimately, 83% of patients would choose outpatient TURB again over inpatient treatment, with 47% having undergone both, outpatient and inpatient TURB.

## 4 Discussion

This is the first study to evaluate the safety and quality aspects of outpatient TURB.

Patient demographics are comparable to bladder cancer statistics, with a median age of diagnosis of around 72 years and a gender distribution of about 6:1 men to women [7]. The distribution of ECOG score is diverse ranging from 0 to 4, reflecting a broad spectrum of physical health and functional capabilities among the patients.

Matulewicz et al. describe an average surgery duration of 34 min for inpatient TURB in their study from 2015 with over 10 000 patients [8]. Our mean surgery time of 11.3 min for outpatient TURB was noticeably shorter, most likely originating from the patient selection process, since patients with tumors ≥ 4 cm and an ASA score ≥ 4 were excluded. While this poses a significant limitation of our study, it is also the core idea of outpatientization of suitable patients, reducing hospitalization costs and alleviating the burden on healthcare facilities. Future studies should explore the feasibility of outpatient TURB in patients with larger tumors to determine if similar outcomes can be achieved.

The rate of non‐malignant findings in 55% of cases likely reflects the decision to exclude patients with tumors larger than 4 cm. This selection criterion aimed to ensure patient safety but might have contributed to a higher proportion of benign lesions.

Concerning the distribution of tumor stages, 91% of patients were diagnosed with non‐muscle‐invasive bladder cancer (NMIBC) ≤ pT1, while the remaining 9% of patients were diagnosed with muscle‐invasive pT2 stage bladder cancer. According to EAU guidelines, ~75% of patients present with NMIBC. Again, the selection process likely was partly responsible for the difference in tumor stage distribution. In a more recent population‐based study by Schned et al. a similar percentage of patients is categorized as NMIBC at 92% [9]. Another likely reason for the shift to less invasive BC findings during the recent decades could be the advancements in medical technology, leading to earlier detection [10].

Resection quality is measured by the presence of detrusor muscle in the specimen. According to EAU guidelines, the absence of DM is associated with a significantly higher risk of residual disease and tumor under‐staging except for pTa low‐grade tumors [2]. Our rate of detrusor retrieval was 58% for all patients and 66.7% for patients with BC other than pTa low grade. DM retrieval rates vary widely in literature observing inpatient TURB, ranging from 49% [11] over 60% [12] up to 67.7% [13], a meta‐analysis by Lin et al. describes detrusor presence in about two‐thirds of all samples [14]. Consequently, detrusor presence in our study is well within a literature range.

In our study, postoperative complications were mostly low grade (Clavien–Dindo Grade 1) including dysuria (52%), transient macrohematuria which resolved spontaneously (28%), and transient urinary incontinence (15%). These results are consistent with literature data analyzing inpatient TURB [15]. Urinary tract infections requiring oral antibiotic treatment, which are categorized as Clavien–Dindo grade 2, occurred with similar frequency (2%) in Rizzo's et al. study [16]. Surgical reintervention because of hematuria, which is categorized as Clavien Dindo grade 3, was necessary for 3 patients (3%). Other authors like Bansal et al. [15] and Rizzo et al. [16] found the risk to be 2%–10% for re‐intervention because of hematuria.

Interestingly, tumor size and surgery duration correlated moderately with higher complication scores, suggesting that the complexity of resection is a critical risk factor; while logistic regression could provide odds ratios and confidence intervals, the low incidence of moderate‐to‐severe complications would yield wide confidence intervals and reduce interpretability, so correlation‐based methods were chosen to include the entire complication spectrum. Future studies with larger samples may apply logistic regression for more robust estimates.

Comparing patients dismissed with or without urinary catheter there was a significant difference in overall post‐operative complication rates. Patients with a urinary catheter may have higher overall complications due to the initial severity of the condition, resection depth, and intraoperative findings that influenced the decision to discharge patients with or without a urinary catheter. This may have introduced selection bias, as patients requiring catheterization could have had more extensive resections or higher risk profiles. Future prospective studies with standardized criteria for catheter placement and comprehensive data collection are necessary to explore the impact of catheter use on postoperative outcomes in outpatient TURB.

Overall, outpatient TURB has a comparable risk of complications compared with inpatient TURB. It must be noted that the absence of continuous medical supervision after patient discharge poses a challenge for detecting and treating the complications in time even if the procedure can be considered as very safe. This highlights the importance of patient education and accessibility to medical staff (e.g., telephone counseling) to facilitate timely presentation in case of emergency.

Bladder cancer recurrence rate was 11.6% within 6 months after initial resection, although all affected patients suffered recurrence at the first cystoscopy after TURB (mean time to recurrence 119 days). Charlesworth et al. provided a median recurrence rate of 18.9% after the first cystoscopy [17]. The higher rate might indicate differences in patient population and tumor biology, this is supported by the fact that the amount of high‐risk NMIBC and muscle‐invasive bladder cancer (MIBC) was also noticeably higher in Charlesworth's publication.

In contrast, a study by Hoogeven et al. found recurrence rates of 7.3% after 6 months when comparing TURB using white light cystoscopy versus TURB with PDD [18]. However, all patients in Hoogeven's study received an SI of mitomycin, possibly contributing to the slightly lower recurrence rate.

Overall, our results underline the importance of postoperative follow‐up, as early recurrence serves as a clinical marker of a poor prognosis and cancer‐specific survival [17].

Patient satisfaction was generally high. This aligns with findings from Lemos et al. reporting high levels of patient satisfaction in a day surgery unit, identifying effective postoperative pain management as an important factor for patient satisfaction [19]. Factors that contribute to patient satisfaction could be the comfort and privacy during recovery around the patient's support network. Furthermore, patients may feel more comfortable not being exposed to unpleasant or potentially harmful situations like noise and sleep disruption, dependence on overburdened hospital staff, or exposure to hospital pathogens. On the other hand, limited access to postoperative care might contribute to anxiety and concerns when complications arise. Also, adequate postoperative pain management can be challenging for inexperienced patients.

However, specific research focusing on patient satisfaction after outpatient TURB is limited, making direct comparisons challenging. Most existing patient‐reported outcome measures (PROMs) in bladder cancer focus more on functional outcomes and disease surveillance rather than on patient satisfaction. Therefore, our study adds valuable data to this under‐researched area.

While our study demonstrates the safety and feasibility of outpatient TURB, it is noteworthy that, according to the German Diagnosis Related Groups Statistics 2024, the mean hospital stay for complication‐free inpatient TURB is ~3 days [20]. The absence of hospitalization of outpatient TURB emphasizes the potential benefits of reducing hospital stays, which can lead to decreased healthcare costs and improved patient comfort by allowing recovery in a familiar environment.

This study suffers from selection bias due to its retrospective approach. To mitigate this, we employed strict inclusion and exclusion criteria applied consistently to all potential participants. Still, the lack of a control group of patients undergoing inpatient TURB prevents comparative analysis and hinders generalization.

Also, patient interviews suffer naturally from recall bias leading to possible inaccuracies and subjectivity, particularly for subjective measures like satisfaction and minor complications. We used a structured questionnaire with straightforward questions to facilitate accurate recall. Shortening the time between surgery and interviews might reduce the effects of recall bias.

Furthermore, the follow‐up period of 6 months could be relatively short, possibly obscuring long‐term efficacy and recurrence rates of outpatient TURB. Longer follow‐up periods would be beneficial to fully assess the oncological efficacy and safety of outpatient TURB compared with inpatient procedures, also investigating topics like progression patterns, long‐term safety profiles, and patient adherence to follow‐up recommendations.

Recognizing the limitations of our retrospective design, future research should focus on prospective studies or randomized controlled trials comparing outpatient and inpatient TURB, incorporating longer follow‐up periods and utilizing validated patient‐reported outcome measures to enhance the reliability and generalizability of results.

## 5 Conclusion

Our study demonstrates the feasibility and safety of transurethral bladder tumor resection in an outpatient setting for suitable patients with tumors smaller than 4 cm. The data show comparable results in oncological and surgical parameters in accordance with guideline recommendations. Patient satisfaction was very high, indicating strong acceptance of the outpatient approach. Therefore, we suggest performing transurethral bladder tumor resection for findings < 4 cm in an outpatient setting wherever possible to reduce the procedure‐related burden for both patients and healthcare systems.

## Author Contributions

**Lucas Strahl:** data acquisition, data analysis, literature research, paper preparation. **Hendrik Borgmann:** study design, proofreading. **Julian Peter Struck:** study design, proofreading. **Johannes Salem:** surgeon, study design, data analysis, proofreading. **Timur H. Kuru:** surgeon, data analysis, paper preparation, proofreading.

## Ethics Statement

This study was conducted in strict accordance with local and national ethical guidelines of the German Research Foundation (DFG) “Guidelines for Safeguarding Good Research Practice.” While this research involved direct patient contact through phone interviews, informed consent forms were obtained from all participants before their inclusion in the study, as per the ethical standards of KLINIK LINKS VOM RHEIN. The phone interviews were conducted solely to gather follow‐up information related to routine clinical care, and no experimental procedures or interventions were involved. All patient data were handled with the highest level of confidentiality and in compliance with data protection regulations, including the General Data Protection Regulation (GDPR). Given that informed consent was properly secured, and the nature of the study posed minimal risk to participants, this study qualifies for a waiver of formal ethical approval under institutional guidelines and national ethical standards.

## Conflicts of Interest

The authors declare no conflicts of interest.

## Supporting information


**Appendix S1** Outpatient TURB questionnaire.

## Data Availability

Data is not publicly available; however editors, reviewers and readers are invited to ask specific questions via the provided mail address.

## References

[1] Cancer Today , “Estimated Number of Incident Cases Germany, Both Sexes, all Ages,” 2020, https://gco.iarc.fr/today/online‐analysis‐multi‐bars?v=2020&mode=cancer&mode_population=countries&population=900&populations=276&key=total&sex=0&cancer=39&type=0&statistic=5&prevalence=0&population_group=0&ages_group%5B%5D=0&ages_group%5B%5D=17&nb_items=10&group_cancer=1&include_nmsc=1&include_nmsc_other=1&type_multiple=%257B%2522inc%2522%253Atrue%252C%2522mort%2522%253Afalse%252C%2522prev%2522%253Afalse%257D&orientation=horizontal&type_sort=0&type_nb_items=%257B%2522top%2522%253Atrue%252C%2522bottom%2522%253Afalse%257D#collapse‐group‐0‐4.

[2] European Association of Urology , “EAU Guidelines—Non‐Muscle‐Invasive Bladder Cancer,” 2023,https://uroweb.org/guidelines/non‐muscle‐invasive‐bladder‐cancer.

[3] DESTATIS , “DRG‐Statistik 2021—Vollstationäre Patientinnen und Patienten in Krankenhäusern,” 2021, https://www.destatis.de/DE/Themen/Gesellschaft‐Umwelt/Gesundheit/Krankenhaeuser/Publikationen/Downloads‐Krankenhaeuser/operationen‐prozeduren‐5231401217014.pdf?__blob=publicationFile#:~:text=Auf%20Basis%20der%20Fallpauschalenbezogenen%20Krankenhausstatistik,Operationen%20und%20medizinische%20Prozeduren%20durchgeführt.

[4] Gesundheitsberichterstattung des Bundes , “Krankheitskosten in Mio. € Für Deutschland,” 2020, https://www.gbe‐bund.de/gbe/!pkg_olap_tables.prc_set_page?p_uid=gast&p_aid=82 280 890&p_sprache=D&p_help=2&p_indnr=64&p_ansnr=65 967 439&p_version=7&D.734=4512.

[5] Bundesministerium für Gesundheit—Regierungskommission Für Eine Moderne und Bedarfsgerechte Krankenhausversorgung , “Zweite Stellungnahme und Empfehlung der Regierungskommission für Eine Moderne und Bedarfsgerechte Krankenhausversorgung,” https://www.bundesgesundheitsministerium.de/fileadmin/Dateien/3_Downloads/K/Krankenhausreform/BMG_REGKOM_Bericht_II_2022.pdf.

[6] P. A. Clavien , J. Barkun , J. N. Vauthey , et al., “The Clavien‐Dindo Classification of Surgical Complications,” Annals of Surgery 250, no. 2 (2009): 187–196. 19638912 10.1097/SLA.0b013e3181b13ca2

[7] Robert Koch Institute , “Cancer in Germany—Bladder,” https://www.krebsdaten.de/Krebs/EN/Content/Publications/Cancer_in_Germany/cancer_chapters_2017_2018/cancer_c67.pdf?__blob=publicationFile.

[8] R. S. Matulewicz , V. Sharma , B. B. McGuire , et al., “The Effect of Surgical Duration of Transurethral Resection of Bladder Tumors on Postoperative Complications: An Analysis of ACS NSQIP Data,” Urologic Oncology 33, no. 8 (2015): 338. 10.1016/j.urolonc.2015.05.011 26072111

[9] A. R. Schned , A. S. Andrew , C. J. Marsit , K. T. Kelsey , M. S. Zens , and M. R. Karagas , “Histological Classification and Stage of Newly Diagnosed Bladder Cancer in a Population‐Based Study From the Northeastern United States,” Scandinavian Journal of Urology and Nephrology 42, no. 3 (2008): 237–242. 18432530 10.1080/00365590801948166 PMC2640838

[10] S. Holmäng , H. Hedelin , C. Anderström , E. Holmberg , and S. L. Johansson , “Prospective Registration of all Patients in a Geographical Region With Newly Diagnosed Bladder Carcinomas During a Two‐Year Period,” Scandinavian Journal of Urology and Nephrology 34, no. 2 (2000): 95–101. 10903069 10.1080/003655900750016698

[11] N. A. Maruniak , K. Takezawa , and W. M. Murphy , “Accurate Pathological Staging of Urothelial Neoplasms Requires Better Cystoscopic Sampling,” Journal of Urology 167, no. 6 (2002): 2404–2407. 11992046

[12] H. W. Herr , “The Value of A Second Transurethral Resection in Evaluating Patients With Bladder Tumors,” Journal of Urology 162, no. 1 (1999): 74–76. 10379743 10.1097/00005392-199907000-00018

[13] P. Mariappan , A. Zachou , K. M. Grigor , and Edinburgh Uro‐Oncology Group , “Detrusor Muscle in the First, Apparently Complete Transurethral Resection of Bladder Tumour Specimen Is a Surrogate Marker of Resection Quality, Predicts Risk of Early Recurrence, and Is Dependent on Operator Experience,” European Urology 57, no. 5 (2010): 843–849. 19524354 10.1016/j.eururo.2009.05.047

[14] L. Lin , X. Guo , Y. Ma , J. Zhu , and X. Li , “Does Repeat Transurethral Resection of Bladder Tumor Influence the Diagnosis and Prognosis of T1 Bladder Cancer? A Systematic Review and Meta‐Analysis,” European Journal of Surgical Oncology 49, no. 1 (2023): 29–38. 35752497 10.1016/j.ejso.2022.06.005

[15] A. Bansal , S. Sankhwar , A. Goel , M. Kumar , B. Purkait , and R. Aeron , “Grading of Complications of Transurethral Resection of Bladder Tumor Using Clavien–Dindo Classification System,” Indian Journal of Urology: IJU: Journal of the Urological Society of India 32, no. 3 (2016): 232–237. 27555684 10.4103/0970-1591.185104 PMC4970397

[16] M. Rizzo , E. Verzotti , G. D. Cosmo , et al., “Perioperative Antimicrobial Prophylaxis for Preventing Infectious Complications After Transurethral Resection of the Bladder: To Use or Not to Use?,” Journal of Endourology 34, no. 2 (2020): 198–202. 31760786 10.1089/end.2019.0523

[17] P. J. S. Charlesworth , R. H. R. Gray , C. Blick , N. Kilbey , A. Protheroe , and J. P. Crew , “Early Recurrence of Non‐Muscle Invasive Bladder Cancer as a Clinical Marker of a Poor Prognosis and Cancer‐Specific Survival,” Journal of Clinical Urology 5, no. 6 (2012): 284–288.

[18] F. Hoogeveen , M. Blanker , E. Cauberg , and M. G. Steffens , “Recurrence of Non‐Muscle Invasive Bladder Carcinoma After Transurethral Resection With Hexaminolevulinate Photodynamic Diagnosis or Regular Cystoscopy,” Scandinavian Journal of Urology 58 (2023): 120–125. 38054524 10.2340/sju.v58.10160

[19] P. Lemos , A. Pinto , G. Morais , et al., “Patient Satisfaction Following Day Surgery,” Journal of Clinical Anesthesia 21, no. 3 (2009): 200–205. 19464614 10.1016/j.jclinane.2008.08.016

[20] Institut für das Entgeltsystem im Krankenhaus , “Fallpauschalenstatistik,” 2024, https://www.g‐drg.de/ag‐drg‐system‐2024/fallpauschalen‐katalog/fallpauschalen‐katalog‐20242.

