# Supplementary material for: Feasibility, Safety, and Patient Satisfaction of Transurethral Bladder Tumor Resection in an Outpatient Setting
Source: Cancer Rep (Hoboken). 2025 Mar 12;8(3):e70120. doi: 10.1002/cnr2.70120 (PMC11897801; doi:10.1002/cnr2.70120)
Supplement: Supplementary file 1 — Appendix S1 Outpatient TURB questionnaire. [file CNR2-8-e70120-s001.pdf]

Outpatient TURB Questionnaire

1.1	Prior to surgery, were you informed that bleeding is a possible complication of TUR-B?	☐ Yes ☐ No
1.2	Did you experience any bleeding after the surgery?	☐ Yes ☐ No
1.3	If yes, was a second intervention required to control the bleeding?	☐ Yes ☐ No
2.1	Prior to surgery, were you informed that an accidental loss of urine is a possible complication after TUR-B?	☐ Yes ☐ No
2.2	Did you experience any accidental loss of urine after the surgery?	☐ Yes ☐ No
3.1	Prior to surgery, were you informerd that experiencing pain or a burning sensation during urination is a possible complication of TUR-B?	☐ Yes ☐ No
3.2	Did you experience any pain or burning sensation while urinating?	☐ Yes ☐ No
4.1	Prior to surgery, were you informed that you might be discharged with a urinary catheter after the surgery?	☐ Yes ☐ No
4.2	Were you discharged with a urinary catheter after the surgery?	☐ Yes ☐ No
4.3	If yes, how did you get along with the catheter?<br>(Please rate using the German grading system:<br>1=Very Good, 2=Good, 3=Satisfactory, 4=Adequate, 5=Poor, 6=Inadequate)	☐ 1 ☐ 2 ☐ 3<br>☐ 4 ☐ 5 ☐ 6
5.1	How do you evaluate the preoperative patient consent talk?<br>(Please rate using the German grading system:<br>1=Very Good, 2=Good, 3=Satisfactory, 4=Adequate, 5=Poor, 6=Inadequate)	☐ 1 ☐ 2 ☐ 3<br>☐ 4 ☐ 5 ☐ 6
5.2	How do you evaluate the TUR-B surgery?<br>(Please rate using the German grading system:<br>1=Very Good, 2=Good, 3=Satisfactory, 4=Adequate, 5=Poor, 6=Inadequate)	☐ 1 ☐ 2 ☐ 3<br>☐ 4 ☐ 5 ☐ 6
5.3	How do you evaluate the postoperative care?<br>(Please rate using the German grading system:<br>1=Very Good, 2=Good, 3=Satisfactory, 4=Adequate, 5=Poor, 6=Inadequate)	☐ 1 ☐ 2 ☐ 3<br>☐ 4 ☐ 5 ☐ 6
6	Did you experience any fear of complications after the surgery?	☐ No ☐ low<br>☐ moderate ☐ high
7	Was there a feeling of being left alone after surgery?	☐ No ☐ low<br>☐ moderate ☐ high
8.1	Would you choose to undergo outpatient TUR-B surgery again?	☐ Yes ☐ No
8.2	Do you have experience with both outpatient and inpatient TUR-B surgery?	☐ Yes ☐ No
